# Facing death, returning to life: A qualitative analysis of MDMA-assisted therapy for anxiety associated with life-threatening illness

**DOI:** 10.3389/fpsyt.2022.944849

**Published:** 2022-09-27

**Authors:** William Barone, Michiko Mitsunaga-Whitten, Lia Osunfunláyò Blaustein, Phillip Perl, Marisa Swank, Thomas Cody Swift

**Affiliations:** ^1^Lucin Psychology Consultation, Oakland, CA, United States; ^2^Kintsugi Psychotherapy LLC, Boulder, CO, United States; ^3^The JFK School of Psychology, National University, Pleasant Hill, CA, United States; ^4^MAPS Public Benefit Corporation, San Jose, CA, United States; ^5^University of California, San Francisco, San Francisco, CA, United States; ^6^Department of Psychosocial and Psychoanalytic Studies, University of Essex, Colchester, United Kingdom; ^7^School of Medicine, Johns Hopkins University, Baltimore, MD, United States

**Keywords:** MDMA-assisted therapy, life-threatening illness, psycho-existential distress, existential anxiety, Interpretative Phenomenological Analysis (IPA), death and dying, psychedelics, 3, 4 methylenedioxymethamphetamine (MDMA)

## Abstract

Anxiety associated with life-threatening illness (LTI) is a pervasive mental health issue with a wide impact. A spectrum of traditional pharmacotherapies and psychotherapies are available, but offer varying success in reducing symptoms and improving quality of life. We explore a novel therapy for this condition by assessing prominent thematic elements from participant narrative accounts of a pilot phase 2 clinical trial of 3,4 Methylenedioxymethamphetamine-Assisted Therapy (MDMA-AT) for treating anxiety associated with LTI. Semi-structured qualitative interviews were conducted with a subset of adult participants 3 months following completion of this trial. This qualitative analysis sought to complement, clarify, and expand upon the quantitative findings obtained from the clinical trial to further understand the process and outcomes of the treatment. Interviews were coded and analyzed using an Interpretative Phenomenological Analysis (IPA) methodological framework. Participants described in detail their experiences from before, during and after the trial, which were analyzed and categorized into thematic clusters. Specifically, participants explored what they felt were important elements of the therapeutic process including processing trauma and grief, exploring mystical and existential experiences, engaging with the present moment with reduced physiological activation, and facing illness and existential fears. Outcomes of the treatment included increased ability to cope with LTI, reduced psychological symptoms, improved vitality and quality of life, and feeling more resourced. Participant narratives also showed a reconnection to life and greater emotional resilience in response to trauma and medical relapse. These findings are compared to similar treatments for the same indication. Limitations and challenges encountered in conducting this study are discussed along with implications for theory and clinical treatment.

## Introduction

The experience of being diagnosed with a life-threatening illness (LTI), receiving treatment, and even achieving remission can be incredibly stressful and traumatic for individuals and their support systems (1). It is not uncommon for individuals at various stages in the treatment process to develop a “chronic, clinically significant syndrome of psychosocial distress” that can present as symptoms of depression, anxiety, or posttraumatic stress disorder (PTSD) (2, 3). These symptoms have been found to occur in nearly half of patients with LTI, and can negatively impact functioning, quality of life, treatment adherence, and prognosis (2, 4). While there have been advances in treating the physical manifestations of cancer and other LTI, more support is needed in helping patients cope with the psychological and existential distress related to these conditions (5).

In recent decades, a resurgence of research into psychedelic-assisted therapies has aimed to provide novel approaches for indications that have traditionally been challenging to treat (6, 7). One area of focus in this field of study has been the exploration of using either psilocybin or lysergic acid diethylamide (LSD) in combination with psychotherapy to treat distress associated with LTI (3, 8–11). In multiple phase 2 clinical trials, researchers demonstrated the potential of a course of therapy aided by either psilocybin or LSD to safely and effectively reduce anxiety and depression in this population (3, 8–11).

Although pharmacologically and qualitatively different from classic psychedelics like psilocybin and LSD, 3,4 methylenedioxymethamphetamine (MDMA) has shown promise as an adjunct to psychotherapy for treating various conditions (12–17). Given the evidence of MDMA-assisted therapy (MDMA-AT) in successfully treating trauma and anxiety-related conditions, as well as its methodological similarities to psilocybin and LSD-assisted therapies, a pilot phase 2 clinical trial was employed to assess the safety, efficacy, and feasibility of MDMA-AT for treating anxiety and other psychological distress associated with LTI (5, 18). The trial assessed participants' scores on a range of standard quantitative measures for issues including anxiety, post-traumatic growth, and depression from before and after a treatment course that included two manualized 8-h therapy sessions combined with either MDMA or an inactive placebo (lactose) (5, 18). Quantitative findings indicated improvement for the majority of participants on primary and secondary outcome measures; however, this was a pilot feasibility study and due to a small sample size was not powered to reliably detect statistically significant between-group differences (18).

While these quantitative outcomes provide important information about the trial, qualitative studies of previous psychedelic-assisted therapy clinical trials have indicated that participants may experience therapeutic mechanisms and treatment outcomes that are not fully captured by the quantitative measures alone (19–21). Quantitative methods are not intended to provide in-depth explorations of participants' lived experiences and perspectives; rather, they focus on quantifiable treatment outcomes (22). Qualitative research has provided an important alternative perspective in exploring psychedelic-assisted therapies, as many aspects of the psychedelic experience and treatment outcomes cannot be properly captured by traditional quantitative measures (19–21).

This qualitative follow-up study was employed to provide an in-depth exploration into participants' experiences and outcomes from the MDMA-AT clinical trial for treating anxiety and other psychological distress associated with LTI. This qualitative study analyzed the 3-month post-treatment follow-up interviews of a subset of participants from this clinical trial (18). These interviews sought to explore the experience of participants before, during, and following this clinical trial, as well as document their perceptions of MDMA-AT as a treatment for psychological distress associated with LTI. The aims of this study were as follows: (1) to complement, clarify, and expand upon the quantitative findings of the clinical trial (5, 18); (2) to identify key themes in participant experiences and treatment outcomes from the trial to better understand the therapeutic process; (3) to identify perceived mechanisms of the treatment leading to sustained reductions in mental health symptoms and/or improvements to quality of life; (4) and assess the similarities and differences to similar treatments that are used for this indication.

## Methods

The individuals interviewed for this retrospective qualitative follow-up study were participants in the Multidisciplinary Association for Psychedelic Studies (MAPS)-sponsored pilot phase 2 clinical trial investigating MDMA-AT as a treatment for anxiety associated with LTI. All of the semi-structured qualitative interviews were conducted 3 months following participants' completion of the treatment. This qualitative follow-up study was approved by the John F. Kennedy University Institutional Review Board and the Western Copernicus Group Institutional Review Board of the parent clinical trial.

The parent clinical trial utilized a randomized, double-blind, placebo-controlled design with an open-label crossover protocol to assess the safety, efficacy, and feasibility of MDMA-AT in 18 individuals suffering from anxiety associated with LTI (5, 18). Participants were recruited through referrals from healthcare professionals, word-of-mouth, and internet advertisements (18).

The study design of the clinical trial consisted of two stages: the experimental stage and the open-label crossover stage. First, the experimental stage of treatment provided identically structured courses of treatment to two groups of participants that included two blinded 8-h experimental sessions with participants randomly assigned to receive either MDMA (*n* = 13) or an inactive placebo of lactose (*n* = 5), scheduled 2–4 weeks apart. In addition, participants received nine 60 to 90-min non-drug psychotherapy sessions; three preparing participants for the first experimental session and three for integration after each experimental session (5, 18). Experimental sessions involved 8-h sessions of manualized MDMA-AT with a male and female co-therapy team, regardless of if participants received MDMA or placebo. Participants' status of being in the randomly assigned MDMA or placebo groups was blinded to both the participant and the therapists. The therapeutic process is described in detail in the treatment manual (5).

Following the experimental stage and the primary endpoint of gathering data from the treatment course, the open-label crossover stage of the clinical trial began. Here, participants in the placebo control group were crossed over to an open-label MDMA-AT protocol where they could receive three MDMA-AT sessions. Participants in the MDMA group were offered one additional open-label MDMA-AT session. At the end of the trial, one participant dropped out due to progression of their illness, leaving 17 participants to receive three full MDMA-AT sessions. The five participants in the placebo group received two additional 8-h placebo sessions and nine additional integration sessions of 60–90 mins over those in the MDMA group. All MDMA sessions included the dose of 125 mg MDMA with an optional supplemental dose of 62.5 mg 90–150 min after the initial dose (5, 18).

### Data collection

A subsample of 10 participants from this quantitative study was selected to be interviewed based on the date of their final drug administration session. The first 10 participants (of 17 remaining) to complete the clinical trial were recruited to participate in qualitative interviews; all 10 agreed and completed these interviews 3 months following study completion. An interval of 3 months was chosen so participants would both have a recent connection to their experience in the trial as well as perspective on outcomes. Participants were informed that this interview was optional and would not affect their involvement in the quantitative study. No financial compensation was offered, and there were no penalties for declining to participate. All volunteers gave their informed consent prior to participation. Of these 10 interviews, two were excluded for technical complications (one recording was unusable due to taking place on a poor internet connection and another interview failed to record). Data collection followed standard practices highlighted in both the literature and in similar studies (19–21, 23, 24). Interviews were conducted through Zoom (an online platform for video conferencing) by a qualitative researcher not associated with the clinical trial, lasted between 1 and 2 h, and were video recorded to later be transcribed and de-identified before being provided to the research team.

Interviews followed the Semi-Structured Interview Guide (Appendix B) developed for this study, which included a number of semi-structured, open-ended questions designed to address the general nature of participants' experiences from before, during, and after the treatment, and allowed space to explore any topics that emerged during the interview. This ensured key topics were addressed while allowing participants to share the unique aspects of their individual experience. Questions included inquiries into participants' lives prior to the study to understand their lived experience with LTI related difficulties, explorations of the phenomenology of the MDMA sessions themselves, and how those experiences were integrated in the following 3 months. Examples of questions include “Can you give me a sense of what life was like prior to the treatment sessions,” “Can you describe in detail your experiences during (this/these) treatment session(s),” and “In what ways do you feel the study has affected your life since the sessions?” (Appendix B). The interview format was designed to allow participants to reflect on the nature and meaning of their experiences in a safe and supportive environment within the convenience of their homes.

### Coding and analysis

Of the eight usable interviews, six were chosen at random using a web-based randomization system, transcribed, de-identified, and sent to the qualitative research team for coding and analysis. Using a model for assessing qualitative study sample size in Interpretative Phenomenological Analysis (IPA) studies (23), as well as the concept of Information Power for developing a qualitative study sample (25), it was determined that a sample size of six would be most appropriate for this analysis. This sample size allows for a balance between providing an in-depth examination of the phenomena from each participant's perspective while also comparing and contrasting between participants.

These interviews underwent coding and thematic analysis by the five-member research team. The research team developed an initial codebook based on their review of the first available transcript. Codes were derived both top-down from interview questions and a model codebook developed for a similar study (19), as well as bottom-up from patterns and themes found in the interviews. Codes were purposely designed to be broad rather than narrow categories. For example, a code of *Psychological Symptoms (Psx)* was used that can capture a wide range of symptom types, rather than having individual codes for specific symptoms of each diagnosis. Using broad codes allows for themes and categories to come directly out of the data, rather than data being pre-categorized by the code structure.

The research team individually coded the first interview, then met to discuss any additions or alterations to the codebook. The research team then finalized the codebook (Appendix C) and developed a model of inter-rater agreement before coding further interviews. The codebook was applied to each interview transcript by a minimum of two researchers using standard practices (19, 23, 26), with the two raters coming to consensus on a unified set of codes before being submitted. Codes and themes were discussed among the team at weekly research meetings to maintain continuity among coders and discuss complicated coding examples. The final codes from each transcript were developed into themes designed to capture the nuances of phenomenological experience from the text of each participant. Themes were then clustered into groups based on commonality and patterns of convergence and divergence. Patterns and connections in themes were analyzed across cases, resulting in a master list of themes for the group. Themes were assembled into multiple categories across time frames of before treatment, during treatment, and treatment outcomes.

A computer-assisted qualitative data analysis software package, MAXQDA 18.2.0, was used to assist in data analysis of the interview transcripts (27). This program helped organize data to identify emerging themes, assess interrater reliability, develop tables of co-occurring codes, and provide an opportunity to easily search through large amounts of data.

### Methodology

This study utilized an Interpretative Phenomenological Analysis (IPA) approach, which was informed by previous qualitative studies of psilocybin-assisted therapy (psilocybin-AT) for anxiety associated with LTI (20, 21) as well as MDMA-AT for PTSD (19). The IPA framework is ideal for characterizing participants' experiences and perceptions of their treatment as it focuses on a *double hermeneutic*: the researcher making sense of how participants make sense of their own experience (23). IPA has been called a “qualitative methodology of choice in healthcare research” and has shown to be useful in analysis of novel treatments such as MDMA-AT as it illuminates the meaning participants make of their experiences in the trial [(22), p.214].

### Participants

The six participants who took part in this qualitative study completed the phase 2 clinical trial of MDMA-AT for anxiety associated with LTI within the previous 3 months and completed both the experimental and open-label crossover stages of the trial. All participants met inclusion criteria for the clinical trial, including having a diagnosis of life-threatening cancer or non-dementing neurological illness that could be ongoing or in remission with a possibility of recurrence, and a prognosis of at least 9 months life expectancy (Appendix A). Seventeen participants had a diagnosis of neoplasms and one participant had a diagnosis categorized as a musculoskeletal and connective tissue disorder. Participants also all scored above 45 (of 80) on the the State Trait Anxiety Inventory (STAI) Trait Subscale, indicating moderate to severe anxiety, which was determined to be secondary to their LTI through a structured clinical interview. The 18 who participated in this clinical trial had a mean age of 54.9 years, 14 were women, and four were men. Upon entering the trial, medical histories indicated that many of the participants were previously diagnosed with anxiety (83.3%), major depression (77.8%), PTSD (72.2%), or insomnia (61.1%).

Of the six participants included in this qualitative study, 100% had a cancer diagnosis in various stages of treatment, and all experienced significant psychological distress related to their LTI. Five of these participants had been in the MDMA group of the trial and one had been in the placebo group, with all of them ultimately receiving three MDMA-AT sessions. All of these participants identified as women, with five identifying as White/Caucasian and one identifying as non-White/Armenian. Participant data for the interviews were de-identified before being sent to the research team, and as such, further demographics were unable to be obtained.

## Results

In their qualitative follow-up interviews, participants explored their experience from before, during, and after their treatment in the clinical trial. All participants (*n* = 6) in this sample described improvements in their mental health symptoms, quality of life, and general functioning, and unanimously credited this treatment process with helping them achieve lasting improvements. Qualitative analysis illuminated what participants felt were important factors relating to psychological symptoms stemming from their LTI diagnosis, the process of treatment with MDMA-AT, and their perceived outcomes of the treatment.

While our complete qualitative analysis explored a range of experiential themes, the following categories were chosen to demonstrate the most frequent, essential elements of participants' experience of the treatment and outcomes reported. Some quotes have been edited to remove filler words, and participants are listed by a de-identified participant number.

### Participant experiences with LTI prior to MDMA-AT

During the interviews, participants recounted their experience of challenges relating to multiple aspects of their LTI prior to participating in the MDMA-AT trial. All participants (*n* = 6) reported that their primary psychological symptoms were a direct result of their experiences with their LTI diagnosis, treatment, and/or remission, and that these symptoms caused substantial impacts to their functioning and quality of life.

### Psychological symptoms associated with LTI prior to MDMA-AT

All participants (*n* = 6) described negative impacts to their mental health immediately upon LTI diagnosis and pointed to the process of LTI treatment as a cause of increased severity of psychological symptoms. In particular, four of six participants described experiencing anxiety and trauma associated with the medical treatment process, and five participants described depressive symptoms secondary to their LTI diagnosis. Three participants described additionally having previous trauma or anxiety related mental health symptoms.

Participant 2 illustrated the sudden impact of being diagnosed with leukemia, recounting that “it felt like someone had kidnapped my life… I felt pretty stopped in my tracks and just in survival mode.” Participant 1 had a similar experience of feeling a sudden and drastic change in her life and immediately struggled shifting roles from being an oncologist herself to being a patient. She remembered, “I went from doing grand rounds two nights before my operation to my whole life changed… It's just having the rug pulled out from under you.”

Four participants recounted how their experiences with ongoing treatments, difficult physical symptoms, and feelings of hopelessness within the medical system led to symptoms of PTSD. Many participants described classic PTSD symptoms of avoidance, re-experiencing, and hypervigilance around their illness. Participant 3 recounted the impact of the initial process of diagnosis and how this had led to ongoing challenges:

There is also PTSD around having a doctor tell me something horrible has happened to me. I mean I had horrible anxiety when I had to go see this neurologist. I just was like “I can't do this, I can't go.” I was convinced I was gonna be told–it seems like I was believing that was all happening to me again.

Participant 1 shared how the process of scans and procedures in her medical treatment led to anxiety and trauma related symptoms: “Just in the anticipation of things I would become extremely, extremely anxious, and just kind of paralyzed, and really avoidant.”

Participant 2 expressed feeling that the trauma symptoms from her medical issues and surgeries could have impacted her prognosis, explaining, “I feel like the compounded trauma had just become stuck and fragmented. That wasn't allowing my body to do the work it wanted to do.”

Four of the six participants were in the remission stage of illness at the time of their interviews, and three described how even this phase of their experience sometimes led to more pronounced anxiety than they had previously in their life or course of treatment. For instance, two participants articulated the experience of feeling stuck in limbo, ambiguity, and a constant state of anxiety around returning illness following their cancer treatments. Participant 3 explained feeling stuck after remission, remembering, “I had these visuals of me being in this kind of purgatory. Like where am I? I don't know if I have cancer or if I had cancer.” She described the impact of this experience:

To have to carry around this demon of death that might strike at any minute. And the thing with breast cancer is it typically comes back in your brain, lung, liver or bones. That means if you have a headache, if you have knee pain, if you have a cough, those could be potential signs of metastasis… I had my first two or three terrible scares, where I got a headache and I rushed to the hospital. And I was like “it's gone to my brain.” I had a couple of those happen to me and I was just like, “I don't know how to do this.” Like I don't know how to move forward from this and just go back into my life.

#### Impacts to quality of life

All participants (*n* = 6) described how their LTI experience significantly impacted overall functioning and quality of life. In particular, participants described notable impacts to their relationships, ability to work, and engagement in meaningful activities. Participant 2 reported, “it affects every area. Like obviously the pain, but your life and how much you're able to work and then your income, my availability as a friend and relationships. The vicarious trauma for my family.”

Participant 6 expressed how deeply she was impacted by her LTI:

I was merely going through the motions of life. And sometimes not even doing that. I mean there was this period where I wasn't even getting out of bed. I couldn't think of a reason to get out of bed.

Participant 3 recounted a similar experience, remembering that the LTI and treatment process felt paralyzing: “I was paralyzed, I couldn't see through it, and I couldn't live my life… I couldn't parent my kids, I couldn't focus on anything. I mean I had a real mental illness, acute, short-term mental illness.”

#### Motivation for participating in MDMA-AT

Participants described coming to the study by varying routes. Multiple participants explained that they needed a different kind of support than what they had previously been offered, feeling that the unique type of existential distress and trauma related to LTI needed a special type of treatment. Participant 3 explained,

I didn't really feel like going to a therapist and like once a week talking about my cancer. That just felt droney and it didn't feel appropriate. To me that feels like how you can deal with normal issues, but this is not a normal issue. This is a big, big thing. And (MDMA-AT) to me felt like a really big, big way to respond to a really big, big thing.

Some participants had read about MDMA being used for PTSD and considered how it could heal emotional wounds. Participant 2 described the need to address her trauma associated with the medical issues and surgeries:

My first intention with going into this study… I needed to disrupt this trauma loop. Because the trauma on top of trauma on top of trauma, you know my nervous system just felt like a five alarm fire happening.

Participant 1 reported hearing accounts of the effects of MDMA and considered how they could benefit from the medication:

What motivated me at that point was this idea I was gonna die not being able to feel or be comforted or trust or heal, like when you have that kind of diagnosis you don't expect to be healed, but there's something in me that wanted to be more whole.

### Experience during MDMA-AT

During their interviews, participants described their experiences during each MDMA-AT session and recounted elements of the experience they felt were pertinent to their process. Participants highlighted themes including the importance of the MDMA experience (*n* = 6), relationship to their illness (*n* = 6), processing trauma (*n* = 5), reconciliation with life and death (*n* = 6), mystical experiences (*n* = 5), reclaiming of presence (*n* = 6), and their relationship with the therapists and study design (*n* = 6).

#### The MDMA experience

When considering her experience with the MDMA-AT in relation to other therapies she had been through, Participant 5 recounted, “Even though I'd been to therapy in the past for different reasons and I'd gone to these support groups and gotten a lot of really great help and insights… this particular experience basically just blew open the walls.”

Thinking about the role of the MDMA in the treatment process, Participant 6 considered,

I think it gets us out of the way. It gets our personality, our story out of the way, so that we can remember what is essentially so. That the oneness of everything is just so. There's nothing that can interrupt that. And we're already whole, as broken as we may be. That wholeness is always accessible. But it's all the stuff, the trauma and the wounding and all the fear and the angst and all of these things that are responses to events, that get in the way. So it helps us to be able to access the truth that's already there.

Participant 1, who had described feeling emotionally isolated through much of her life and having a hard time connecting to or sharing emotions, recounted that during the MDMA-AT sessions, “It's just like it all came out. One story after another, patients, kids, and dying. All the things that had ever stuck to me were there for me to talk about. So, it was a really profound experience.” She explored what it felt like to help get to that place:

Fear was gone. Just the constant fear of the whatever must be, the overdrive, or the abnormal. That sense of fear being triggered or taking over. And that was gone. It was like I was looking around, like not outside, but I was looking around in my brain, in what's created out of the brain, you know, like the physical sense of it. So not just like how trauma works, but how everything works, like I had a feeling or an experience that I could see how everything works. Everything I've ever studied, or know about, or been exposed to, I could see everything worked and how it all fit together. It was an incredibly expansive experience, and I can't say it was an intellectual experience, it was like a mind experience. But the vehicle was my body. People ask me if it's spiritual. How could it not be, right? But it was also coherent and it was logical.

Participant 2 described that coming into the MDMA-AT sessions, she could sometimes see her “East Coast Work Ethic” come in, feeling that she had to work hard to get the absolute most out of the sessions. She remembered telling herself, “Okay, now you have to get to work and run each of those traumas through. Like we're gonna heal each one of them individually.” She remembered that in response to this, “The medicine kept saying, ‘No you don't, it doesn't matter. It doesn't matter.' And I felt such relief in that. This is the healing. Just allowing yourself to be here.” She remembered, “this was a big theme to the worker bee in me, there's nothing to figure out. The words that kept coming through all the sessions were trust and allow, just trust and allow it to happen.”

Participants also described going into the MDMA-AT sessions feeling that they were supposed to focus completely on their illness, death, and existential issues, given the focus of the study. However, while portions of the sessions did focus on those issues, the non-directive style of the treatment also allowed for other important content to arise during the sessions. All participants (*n* = 6) reported that allowing for focus on other issues from non-LTI trauma to attachment issues was also integral to their healing process. Participant 3 illustrated this:

I've had all these other things that came out of it that I didn't have any intention around addressing at all. I didn't even know they were issues. I feel like I've just gotten so much from it. It's such a gift, such an extraordinary gift.

#### Relationship to illness

While the interviews showed that participants' relationship with their LTI shifted in the long term, all participants (*n* = 6) also described that relationship evolving in real time during their MDMA-AT sessions. A notable example of this process occurred for Participant 2, who expressed an important realization in one of her MDMA sessions that her LTI was not the entirety of her identity. She recounted a feeling that “It was really clear. My health challenges were not defining me.” Participant 3, remembered having an illustrative vision during an MDMA session and said, “Behind me is the cancer and it's this thing in the past. It's contained back there. If I look and turn around, I can see it. But it's almost fortified because it ended. It's a thing that's stopped.”

Participant 5 also had a momentous realization during an MDMA-AT session about how she could relate to her cancer:

The biggest thing that came up, and it was huge… my big epiphany was that I wasn't going to die from cancer. Something that wasn't going to kill me anymore. Like all along I was like “what have I done?” and “what am I doing?” Like I made this happen. And just being afraid of eating a little cookie or something, because sugar feeds cancer, right? Or having a drink, you know, not taking that pill that I had to take everyday and stuff. I got a real sense of my body and where it's weak and what I need to work on. But I just knew that the cancer wasn't going to kill me. It wasn't going to come back and kill me.

Participant 3, who was actively in remission but experienced considerable anxiety about cancer recurrence, talked through how she sensed her body during an MDMA session that led to a positive change in perspective:

The first thing that happened was I did this body scan. So I went into my body and I traveled through it and started at the tip of my toes and circled around each of my little foot bones and in and out of the tendons and the tissues… I could see it so clearly. And I could see the colors so brightly. They were just like bright blue and red. I traveled all the way up through my legs, like through the area where my ovaries used to be… through my liver and my lung and stomach… and it was all so healthy and vibrant and my life force was just like sparkling. Literally there were sparkles… I finally went up to my beautiful brain and it was so clear. There was no cancer anywhere and there was just health.

#### Processing trauma

With the clinical trial focusing on anxiety associated with LTI, many participants expressed surprise at how much of their sessions ended up naturally focusing on trauma processing. All participants (*n* = 6) described traumatic experiences related to their LTI diagnosis, treatment process, and/or experience of remission, and three participants also shared past traumas that were unresolved at the start of the study. Each participant described how the study allowed them to process compounding emotional wounds in their lives from their LTI and beyond. For example, Participant 4, who experienced many traumas throughout her life as well as from her LTI, described how accessing traumatic memories was more achievable with this treatment than her attempts with regular talk therapy:

The connection I felt with previous traumas and health things was also palpable. Which is something that you don't get in talk therapy. It just doesn't happen. Very, very uni-dimensional compared to the multi-dimensional aspects of the MDMA therapy… I was able to talk about traumas that I had experienced earlier in my life, that I had not been able to talk about at all before…What happened as a result of the second session is that I said to Therapist 1, “I think you wrecked my [trauma] story,” which is not a bad thing because it wasn't a good story, it wasn't one I wanted… I knew at the end of the third [session] that I no longer had PTSD and it was gone. It was just gone… A huge, huge burden had been released. I had been carrying that around. It had just been so awful to hold that inside. Letting go of that story just freed up a lot of energy. It was truly amazing, truly amazing, and completely unanticipated.

Participants described how MDMA-AT allowed them to confront and make meaning of their traumas in a new way. For example, Participant 1 discussed how during her MDMA experience, she was able to confront memories and emotions related to a near-fatal sexual assault which occured over 20 years prior. She was explicit in exploring how her early traumas contributed to the anxiety she felt when diagnosed with an LTI, and was able to differentiate them:

We went right into my past, childhood, early adulthood, a big trauma I lived through. And then it started to become way more clear why I had made all these connections and the puzzle of my anxiety became very clear, like the origins of it. And it quite separated itself from the cancer experience.

She continued,

I had never had that experience before, where I could just sustain, trust, and listen and tell the whole truth or emotional truth or say exactly how I was feeling. That was MDMA, for sure. I would not have ever been able to do that. I had never really told the story [of my assault] to anyone–I didn't have access to those memories. They were more like a distant story. Like I knew the content, but I had never really had access to how I actually felt when it was happening. So, the second session was a big breakthrough.

Referring to her process of facing the trauma of battling cancer during her MDMA-AT sessions, Participant 2 considered, “It's not like [MDMA] erases a memory, but it takes the claws out so it doesn't own you anymore.” Explaining her experience of how the MDMA contributed to her ability to process this trauma, she remembered the medicine “allowing the body to have experience that contradicts anxiety and trauma. That's really what I had… these affirming experiences are deep and visceral.”

Arguably the most important factor of trauma-informed psychotherapy is for patients to feel a sense of safety (28, 29). Participants in this study pointed to feelings of emotional security during the treatment as an aspect of MDMA-AT that helped them come to important insights and process trauma. For instance, Participant 5 described the treatment: “like Heaven in a way. It was like, I felt so safe. In such a safe environment that I realized how unsafe I felt most of my life.” Participant 2 recounted this importance of safety in her process, remembering, “that was kind of a big theme for me, feeling safe in my body. That's what I experienced with the medicine. For the first time in years and years and years.” Participant 1, who had described considerable challenges with being vulnerable and trusting others through her life, looked back on her sessions and reported,

I took the MDMA and that trust, I can even feel it now, that feeling that the fear is gone and the trust and the desire to connect with people just came online. And then the rest of it is like working out the details, pretty much.

#### Reconciliations with life and death

All six participants said that during the MDMA sessions they were able to interact with and consider the concept of their own death without the fear, anxiety, and dissociation that they had previously experienced. In one example, Participant 3 was able to utilize the feelings of emotional safety occasioned by MDMA to fully confront her fear toward and accept the reality of death, reporting:

While I was in [the MDMA session], I realized that I could consider my death. Like I could go in there and consider it. So as soon as I had that idea, I was like, “Okay, I want to talk to them about this.” So I sat right up and I was like, “Okay, let's talk about this. What would happen if this cancer came back and I died?” And we just had this whole conversation about it. And we got to the fear, like what was the worst thing about that scenario?… “Okay, my kids, I'm giving them so much. I've given them so much, and they are really gonna be okay.”… I realized I didn't have a ton of fear about me not living anymore… not that I want to die, I love life.

Participant 1 also described coming to a deeper understanding and comfort with the concept of death from the expanded and non-reactive perspective offered by MDMA:

It wasn't drama. It was just the human condition, and illness, death. I was seeing my life-long inquiry into life and death from this very expanded place. And it's as if I had these invisible lines of architecture of how consciousness works.

Participant 2 described feeling a deep acceptance with the potential of death: “I remember feeling after the last session if I died tomorrow, which I'm not invoking at all, but like I would feel happy and complete in this.” She considered a notable feeling from the sessions that contributed to this sense. She remembered, “there was a real, real sweetness. The messages I was getting were like a Mother Goddess thing, like, ‘I got you, you're okay, you're gonna be okay.' I remember just saying, ‘I'm okay, I'm gonna be okay.”' The authentic acceptance of death she experienced in the trial led her to feeling alive again: “I just felt like in a lot of ways I was feeling more hopeful and excited and I guess had a reset button on life. Just feeling alive after not is pretty amazing.”

#### Mystical experience

While research on psilocybin-AT shows that having a mystical experience (ME) during the session is correlated with producing positive outcomes (21, 30, 31), previous MDMA-AT studies for PTSD suggested that ME was not correlated with symptom reduction (19, 32). ME also appears to be less prevalent in the use of MDMA than in psilocybin or other classic psychedelics (33–35). However, all but one participant (*n* = 5) in this study reported that some dimensions of ME were present in their sessions and felt these experiences may have played a role in the process of healing. Prominent ME descriptors from the study data include feelings of unity and interconnectedness with all people and things (*n* = 5), a sense of peace and joy (*n* = 4), a sense of sacredness (*n* = 5), ineffability (*n* = 5), an intuitive belief that the experience is a source of objective truth about the nature of reality (*n* = 5), and a sense of transcending normal time and space (*n* = 5).

For example, Participant 2 described a sense of unity while experiencing the medicine, feeling she was “being fueled by everything. The whole interconnectedness piece. I know that people talk about meeting a divine more with psilocybin, but this was absolutely like a million percent me being reconnected.” Participant 5 also described a sense of universal merging, remembering, “My body became, it was almost like it was part of everything. It's like to become, like I am the stars and the sun and the trees.” Similarly, Participant 6 explained, “I became the earth, giving in this really abundant way that the earth gives us…. All these different people and things that I was. I was a hummingbird and I was the orchid.” She considered the connectedness she felt from this experience, “it was a huge exercise in walking in someone's shoes. And really taking that to heart.”

Participant 1 described her experience of altered perception and awareness:

I was aware I was in a state of consciousness very much grounded in the witness. And I saw all the dimensions of the transpersonal state and different states and stages of consciousness. And I wasn't traveling through them but it almost expanded to fill the whole house and it was my mind. And I retained my autonomy.

While not commonly expressed in a previous qualitative study of MDMA-AT for PTSD, multiple participants in this study described transcending space and time in their MDMA sessions (19). Participant 1 vividly recalled an illustrative experience during her second MDMA session,

I went through a sense of breaking through into a very warm feeling in my chest. And that was quickly an entry into the very cavernous, spiritual state. And then it was just a series of traveling. It was a physical experience. It just was like I was passing through gateway after gateway. It had the sense of going down, like sort of feeling everything deeper, but it was all centered here. And then I would enter into different realms, and I decided to call them that. Very visual, very vivid. And it's like you could spend lifetimes there, it was incredibly beautiful.

While ego boundaries seem to be loosened, the concept of self did not appear to be entirely absent in the MDMA experience. For instance, Participant 1 explained, “So I was ‘I' throughout the whole thing. I could see myself, I could hear myself. They ask lots of questions about experiences of oneness. I mean it was definitely an experience of oneness and connectedness.”

#### Reclaiming of presence

When describing their experiences prior to the clinical trial, many participants (*n* = 5) described various aspects of feeling separated from the present moment, either through dissociation, avoidance, or perseveration on the past or the future. Participant 3 illustrated this experience in remembering “feeling caught in the past and the fear of the future, and not living in my present moment” before MDMA-AT. During treatment, all six participants reported feeling a reclamation of the present moment and a reconnection to self and life.

For example, in recounting a moment during an MDMA session, Participant 2 shared that she was able to connect mindfully to the present, remembering the feeling of “finally feeling what it feels like to be in the present and not the past or the future… I felt like all of me is here, if that makes sense. Whereas I felt like I was so fragmented before.”

Similarly, Participant 5 described how the MDMA helped her to come into presence with herself:

Just trying to love, or be compassionate right now instead of worrying and thinking about what might happen. Being everywhere else but right here. And that's what the MDMA helped me with, was to bring me right back to myself.

Participant 3 felt the spiritual nature of having the ability to truly engage in the present:

The being present piece was just, the emotion that I have with that, is sort of like euphoria. Like I feel so much awe. You know, it feels divine to me. It feels very spiritual and divine, more than emotional. Under the MDMA, I went to this visual place of–and this is so clear for me, this very clear and open plain. With light pouring down on it, which is my present space. And it's just, it's me and I'm healthy.

#### Experience of the study design and therapists

In their interviews, participants also considered how the treatment design contributed to a process of healing. All participants (*n* = 6) expressed an understanding that the MDMA is only one part of the system, and that without the other parts of the treatment, it may not be as beneficial. Other important aspects of the treatment process that participants described were the trusting relationship with the treatment providers (*n* = 6) and the container of the study design (*n* = 6).

Participant 4 highlighted the importance of the treatment design and therapists, explaining, “the intensive 6 h with two therapists, that was a tremendous opportunity to go very deeply as well.” She added, “I know MDMA created that safety, that was so essential. I loved the way the study was set up. I thought it was so perfect, that there was time to process in between, and to establish a relationship initially.”

In describing the deep emotional connection Participant 4 felt with the therapy team, she expressed,

The connection I felt was a significant part of why I think it was effective. The connection I was able to establish with [the therapists] and them with me, was very deep and very spiritual and very loving and palpable.

Participant 1 also reflected on the importance of the factors beyond just the medicine, explaining,

It's a triad that was critical for me. The very tight container. A very controlled environment. And then the second part was the skill of the therapists and the devotion. And then the third piece was the medicine. And I would say that the connectivity with the people was as important if not more important than the medicine.

Continuing to describe the importance of the therapeutic relationship and the impact this had on her experience, Participant 1 recalled of the study therapists, “I felt their dedication and I could see it, and obviously I benefited from it and we developed a very close bond. I think for someone like me, they were the perfect guides.”

Participant 3 felt the therapist team emulated parental roles that she felt were important to her healing process:

They were very Mom-Dad for me. Like she's very nurturing and kind of holds space for me emotionally. And then [he] would kind of have this ridiculous sharpness and he would come out with these things, and I would be like, “Oh my God you just blew my mind,” like over and over.

### Treatment outcomes

Qualitative analysis of these participant interviews illuminated a range of outcomes from the treatment that complement and expand upon the results of the small pilot clinical trial. As was displayed in previous qualitative explorations in the psychedelic sciences, many important outcomes can be difficult to discern from quantitative symptom scores alone, as these treatments involve complex processes of growth and change that can expand beyond symptom reduction (19). Exemplifying this is that the entire sample of participants (*n* = 6) credited this treatment with significant positive changes to their life, while the quantitative outcomes indicate mixed results on outcome measures (18).

Participant 2 illustrated this change by telling, “I can gush on for a long time about the depth of my gratitude… and how much it's given me back my life. But not the same life as before, but in probably a deeper way, even.” Participant 6 also reflected an experience of feeling the MDMA-AT allowed her to get her life back and feel like herself again:

There's no way I would be where I am right now without this medicine. I would not be. So it feels like it's given me back my life. Not that old life, because that old life is gone, but it's given me back, me.

Participants described qualitative improvement in their Management of Medical Symptoms and Relationship to Illness (*n* = 6), Psychological Symptoms (*n* = 6), Vitality and Quality of Life (*n* = 6), and Self-Awareness (*n* = 6). Preparatory and integrative therapy sessions, strong rapport with the therapy team, and the beneficial effects of MDMA in the treatment process were perceived as factors in aiding long-term benefit.

#### Management of medical symptoms and relationship to illness

All participants (*n* = 6) reported that they experienced considerable challenges in coping with medical symptoms and their illness trajectory prior to MDMA-AT. Each participant described an ever-present awareness of their illness that impacted their mental health, identity, and functioning. These participants explained how they witnessed the ways in which they related to their illness evolve throughout their experience of the trial. Regardless of the trajectory of their illness, each participant described improvements to how they managed their medical symptoms and related to the illness following the trial.

Five participants described that prior to the clinical trial they became hypervigilant to medical symptoms, with physical discomforts such as headaches sometimes triggering panic attacks or hospital visits. Participant 3 described a change in her emotional response to physical symptoms that were unlikely to be caused by cancer:

I think in a different time I would've been anxious about that. And I just really—I really wasn't. I was like, it should pass, like it usually does. If it doesn't pass, then again, that's something I will have to face when it doesn't pass. Which is something that would be in my future, which is not happening right now. So I just was able to kind of like, really put the re—not even reframe it, just frame it.

This was echoed by Participant 1, who noted a shift in her response to medical symptoms and treatments as well as a reduction of anxious perseveration on health issues:

I still have to handle the neuropathy and the pain at night and stuff like that, and I've got issues, of course radiation. But it's just not taking up a lot of real estate. I'm not so focused on those problems. I think the anxiety was making me glom onto them before. So now I think I'm just a little healthier about my attitude to it.

At the time of her interview, Participant 2 continued to face challenging medical symptoms and treatments related to her cancer. She noticed how she was managing these events differently following the MDMA-AT trial:

I feel really strongly that even though I'm having some [cancer] symptoms, and they have to do treatment, that the healing from the [MDMA] sessions is still happening. I can't emphasize that enough. Like I can feel it. Even in the midst of symptoms that are causing me pain or discomfort, or make me scared or whatever, I feel very resourced. The healing is still happening.

Prior to the trial, many participants described how their medical issues became a large part of their identity. They felt that much of their world revolved around dealing with symptoms, going through treatments, or focusing on the challenging psychological and existential aspects of the experience. Participant 6 described that even amidst continued medical treatment, she noticed a very different relationship with her illness:

It feels like the cancer is something that happened to me… I'm not paying a whole lot of attention to it. I'm just not. I'm still on hormone therapy and I'm supposed to be on hormone therapy for 5 years. I'm living my life. I just don't pay a whole lot of attention to it… [it's] no longer consuming me or defining me.

Participant 4 talked about how the change in relationship with her illness allowed her to have hope about living that was previously impacted by fear. She felt this changed her intentions in life altogether:

The hope that I have, and the lack of fear about living. My intention now is to live, and to live a good life. And I feel confident that I can do that. Whereas before I didn't believe that at all. And I think it would have affected my physical health.

#### Reduction in psychological symptoms

In addition to meeting criteria for moderate to severe anxiety as inclusion criteria for the clinical trial, participants described a range of psychological symptoms that stemmed from their illness and process of treatment (18). Four participants shared they were also struggling with mental health issues that pre-date their LTI when the study began. All participants (*n* = 6) described that through the course of this study they experienced a noticeable reduction in psychological symptoms related to their LTI as well as other issues.

In considering her experience of change in this treatment, Participant 1 reflected, “the focus on the cancer anxiety and that whole thing is completely resolved, and even more than I would have hoped for.” After previously having debilitating anxiety and trauma symptoms related to medical issues, she felt her nervous system was no longer overactive:

I don't have an internal reaction where I'm clenching and just super uncomfortable and all that autonomic activity going on, or shame, or limbic hijack, or however you guys say it, is not happening anymore. That's definitely MDMA, because that's a big shift.

Participant 3 described a very similar experience, recounting, “I feel like when I hit moments where I used to struggle, I'm able to navigate them without getting anxious, so I really do feel strong.” She spoke about noticing a change in her anxiety response to having a cancer scare:

It just didn't bring me any feeling of chest tightening at all. So I feel like that chest tightening, adrenaline rush pattern is just not—I just don't feel it happening. I feel like I stay in a calm place while I kind of frame it. And then it's framed, so I don't need to accelerate…Reframe, stay here, not happening right now, if it happens, that would be down there, not now. And then I just stay down here.

Five participants described debilitating issues with depression either before their LTI or as a result of it and saw changes to their symptoms of depression in moving through this treatment. Participant 6, who said that she was previously “merely going through the motions of life, and sometimes not even doing that,” explained her current experience of depression:

Gone. It's gone. It's gone. The depression is gone. And that doesn't mean that I don't have stuff going on in my life that troubles me, but it feels like the normal way that I've always been with stuff. Sitting here now, it felt like some alien energy invaded my life and came in and took over my life. And those aliens are gone.

Participant 4 also considered how she could be impacted if her depression did not improve:

Had I gone on to be as depressed as I was, if I hadn't actually taken my own life, it wouldn't surprise me if the cancer came back. Whereas I think the chances of that happening are lessened significantly by the change within me.

Many participants expressed a clear recognition that while these improvements are present, that in most cases continued work on these long-standing psychological issues needs to be done. Participant 1 expressed this around the long-standing trauma work that was uncovered and worked on in the MDMA-AT sessions:

Uncovering that earlier trauma, I think I have work left to do with that. And that I can see how it shows up, it's a lot of digesting for decades of the effects of it. So, that is not finished, but I wouldn't have had that as an expectation, that it's finished. But I feel it's very workable now. In a way that wouldn't have been possible before.

Participant 6 described her experience of ongoing anxiety and panic, but how her response to these symptoms has dramatically changed following this trial:

The anxiety, I did actually have a little panic attack at one time. It's the only one that's come up since. And I was removed from it, I watched it. I said, “Oh, it looks like I'm having a panic attack right now.” And then right after I said that outloud, “No, I don't think that's gonna happen.” And then I just watched the whole thing, I felt the whole physical manifestation of it. And then watched it go.

#### Improved vitality and quality of life

All participants (*n* = 6) described the ways in which their LTI was a detriment to their quality of life, impacting social relationships, mental health, energy, passion, and vitality. They all report improving their overall quality of life and feeling more able to embrace life once again as a benefit of this treatment. Participant 1, for example, shared, “the knowledge that I was alive was very solid. And I wanted to be alive and I had this big life-force in me.” She felt more connected with people and the intrinsic vitality within her:

I took the MDMA and that trust, I can even feel it now, like that feeling of the fear is gone and the trust and the desire to connect with people just came online. And then the rest of it is like working out the details.

Participant 2 also felt more connected with the people in her life and reported that they took notice in her sense of passion and engagement with life following MDMA-AT:

I mean my family and friends after that, like I felt better after that session than I had felt in years and years and years. And everybody noticed. Energetically, what I had said, my level of engagement, my level of aliveness and passion. And, yeah, everybody noticed.

She realized that MDMA-AT helped her gain access to an aliveness she has not felt since before her LTI diagnosis, recalling: “I'm feeling more alive than I have for years and my experience with the medicine is helping me access my bright spirit and the wild mind that came through me as so much bigger than the pain.”

Similarly, Participant 4 noticed feeling much more able to engage in relationships as well:

I feel so much more social. I have had a lot of great conversations with my friends and family and my relationships with them have gone much deeper. And I've felt absolutely like a different person in terms of my ability to get in touch with how I feel and communicate that. It was something I just could not do before, I didn't know how I felt.

Participant 4 continued on to share how she feels a renewed sense of energy and happiness following MDMA-AT:

I felt physically lighter. I had so much energy I don't know what to do with it. I'm so happy. I mean I used to be involved in all kinds of things. And I'm back to that again, after all those years when I was incapable. So, it's real clear. I'm even able to sleep well. And I had not been able to sleep well for 20 some years.

#### Feeling more resourced

Inspired by feelings of safety and clarity occasioned by their MDMA sessions, all participants (*n* = 6) describe palpable ways they have incorporated new strategies of coping into their daily lives. For instance, Participant 1 gained a deeper understanding of her defenses and ways she can utilize new capacities for coping:

It was like hitting at the very core body memory, and through that process, and now through these new capacities I have because of the MDMA, I can kind of do it on my own, figure out the different components of why I have so much anticipatory anxiety and why I will avoid.

Participant 3 also spoke to this improved ability to manage anxiety as it connects to an in depth understanding of her psyche, expressing, “I feel like when I hit moments where I used to struggle, I'm able to navigate them without getting anxious, so I really do feel strong. But, I also didn't know all the layers that were there before.” She also felt she learned the important skill of acknowledging her health in the present moment following the treatment:

When I get stuck thinking about the cancer that happened, I'm like “I'm not there, I'm here.” Or if I think about a fear of recurrence, I'm like, “it's not happening, it's not happening right now.” And so, yeah, so that was the big takeaway.

Many participants expressed understanding that the treatment did not change their life circumstances or challenges, but rather gave them new ways to move through those things. Participant 4 explained feeling,

…the ability to address my problems so much more effectively. Yeah it wasn't magic—the pill didn't make my life circumstances go away, but it enabled me to access all of the resources that I have. And just deal with the ups and the downs and see a way through for positive outcomes in every area of my life.

Participant 2 reiterated this as well, remembering how overwhelming her trauma symptoms could be and how much more able to cope with life's difficulties she feels now. She explained, “there's still stuff I'm dealing with and having to deal with, but like I said I'm feeling so much more resourced compared to when I was in the trauma loop I would get flooded pretty quickly.”

#### Improvements in presence and self-awareness

All participants (*n* = 6) reported challenges in feeling separated from the present moment and many aspects of themselves through their LTI journey. MDMA-AT appears to have broadened participant perspectives and allow them to look at their life and situation with increased self-compassion and self-awareness. For instance, Participant 2 remarked on the wholeness she felt following MDMA-AT:

I felt like all of me is here, if that makes sense. Whereas I felt like I was so fragmented before. And another thing that's more recent, because I mean I still feel the healing happening on so many levels.

Participant 4 also felt a deep sense of integrity that evolved with each MDMA-AT session:

It was like the guilt and the shame lifted, you know, this huge weight. I have to say, after each session, even the second one, I felt relieved, very relieved, excited and thrilled. Each time even more so. And at the end of the third session I thought I felt complete.

Participant 4 continued on to share about how her trauma no longer defined her sense of self, remembering, “I would take in traumas and difficult circumstances before and make them part of my definition of self, you know. I'm not doing that at all [now]. I'm really clear about who I am.”

Participant 3 also noticed how becoming more aware of her internal process in the moment has changed the way she interacts with herself and the world:

It's not happening right now. It seems hard to understand, you know, now that I'm saying it. But it just became so clear. It became so true. And that truth changed me. You know, it changed the way I am in the world and the way I orient myself from the thing that had happened and the things that might happen. Cancer or no cancer, that is such a gift. To really be able to be in the present moment is just such a gift.

## Discussion

In the first clinical trial of MDMA-AT for treating anxiety and psychological distress associated with LTI, results indicated that many participants showed improvement on a range of primary and secondary outcome measures, the treatment was well tolerated, and no Serious Adverse Events (SAE) were reported (18). In this pilot study however, not all participants showed significant improvement on outcome measures and the small size of the study lacked the power to find statistically significant results (18). In this qualitative follow-up study, a subset of participants from this trial all described experiencing considerable changes in their relationship to LTI, improvements to functioning and quality of life, and reduction in mental health symptoms. The lessons from this qualitative study are important to further understand the experience and outcomes of participants within this trial, as well as to explore the possible mechanisms of MDMA-AT for this indication and how they relate to similar psychedelic-assisted treatments.

### Possible psychological mechanisms of action

Multiple participants described that while the intention of this treatment was to focus on anxiety around LTI, they spent much of their sessions focusing on other topics altogether. Some participants expressed feeling concerned at some points that they were not focusing on topics of illness or death as they expected they would, but experienced reassurance both from the therapists and the medicine to follow the process where it goes and trust in their *inner healing intelligence*. In this subset of participants, processing trauma was a particular focus throughout their treatment. This included processing trauma around their health journey, but also focused on old wounds that continued to cause issues in their lives. Additional psychological mechanisms included mystical-type experiences and existential exploration, engaging with the present moment, and facing the illness with an objective and compassionate perspective.

#### Processing trauma

Five of six participants in this study described that an important factor of their healing was processing the traumas that arose throughout their process with LTI, as well as their life before. MDMA-AT has shown to be particularly well suited to aiding trauma-focused therapy, leading to posttraumatic growth (13, 14, 36, 37). Potential biological mechanisms for MDMA to decrease PTSD symptoms have been explored and include MDMA's effects of reducing amygdala activity and increasing activity in the Ventromedial Prefrontal Cortex (VMPFC), which reduce common symptoms of PTSD during the treatment and can allow for exploring the traumatic memories without the associated physiological response that can impact the process (37–39). These and other neurobiological effects in combination with a trusting therapeutic relationship and safe container appear to allow participants to feel emotionally safe enough to process traumatic material that is otherwise difficult to access (19, 39–41). When trauma is emotionally tolerable to confront in therapy, it may become possible to deal with traumatic elements following diagnosis and treatment of an LTI. This mechanism of action indicates one major difference in how MDMA-AT treats anxiety associated with LTI differently than psilocybin-AT.

#### Mystical experience

Research shows that other psychedelic medicines, such as psilocybin and ketamine, tend to evoke a wide range of ME. These can include a *sense of unity with all in existence, a sense of sacredness, deep feelings of peace and joy, and ineffability* (3, 21, 42). Studies of psilocybin-AT have also found the level of ME to be a key indicator of positive outcomes in the treatment (43). While a previous qualitative study of MDMA-AT for PTSD showed participants experienced a limited range of ME and that there was no correlation between level of ME and outcomes, multiple participants in this study described significant experiences that qualified as ME beyond what is commonly seen in MDMA experiences (19, 32). The most prominent ME descriptors outlined by participants in this study were *interconnectedness* and *transcending of normal time and space*. For these participants MDMA-AT decreased intense feelings of isolation and existential loneliness, which can be common experiences associated with LTI. Temporarily feeling connected to all things and loosening one's orientation to time and space may help those living with LTI or in remission feel able to be more engaged in the present and value connection with others in a deeper way. Participants also described feeling that in MDMA-AT their MEs remained grounded and connected to self, rather than dissociated or characterized by ego dissolution. This may have supported these important experiences in feeling grounded and real, potentially aiding in their ability to be integrated into a long-term understanding beyond the sessions themselves.

#### Engaging with the present moment

A common experience in depression and anxiety disorders is difficulty engaging with the present moment (44). In these conditions, focus is often directed to either the past, where things may have taken place that can no longer be changed, or into the future where there exists the unknown and feelings of lacking control (45). For people experiencing challenging situations in life including illness and pain, spending time in the present moment could be especially difficult. Multiple participants in this study described how their relationship to the present moment evolved through the treatment process, and how this played an important role in how they went on to cope with their situation more effectively. While engaging with the present moment, participants described deeply understanding that they were not currently dealing with the unknowns that led to anxiety, namely recurrence/worsening of illness or death. This was often paired with increased confidence that they could deal with those things when/if they did arise in the future. Participants described the importance of both the MDMA and the therapeutic container in supporting their process of engaging with the present moment. In the optimal arousal zone occasioned by this treatment, participants can practice dealing with the discomfort that may exist in the present, and relearn their capability of managing what they find there.

#### Facing illness and existential fears

Participants described altering their relationship with illness and the life/death process as another integral part of this treatment. Participants described that MDMA-AT allowed them to re-identify with being their own person separate from the illness, and also gaining perspective on their feelings about living and dying. Having an altered perspective, increased compassion, and decreased fear and anxiety during MDMA sessions allowed participants to calmly and objectively address these challenging topics and identify their feelings beneath the fear and anxiety, which led to lasting changes in these perspectives.

#### Comparisons to psilocybin-AT for LTI

While psychedelic treatments for psychological issues associated with terminal illness have for decades focused on psilocybin and LSD, the parent clinical trial was the first exploring MDMA-AT for this indication (3, 8–11, 18). This provides a unique situation where the phenomenological similarities and differences of psilocybin-AT and MDMA-AT can be explored for the same indication. Despite their differences, there were remarkable similarities in the phenomenological descriptions of healing outcomes between MDMA and psilocybin, including reconciliations with death and dying, acceptance of cancer in one's life, reduction of psychological symptoms, and improvement in quality of life.

While there are similarities in the treatment structures, each of these medicines appear to reach their outcomes in quite different ways. For example, psilocybin sessions commonly include long periods of internal processing where a participant may experience vivid visualizations, alterations in their sense of self (sometimes including complete ego dissolution), and/or wide ranges of emotional experiences (20, 21). These experiences can be rich with relational, autobiographical, spiritual, epistemological, and ontological material that aid in exploring factors of their life and the concepts of life and death (20, 21). Some studies have found that more than 50% of participants experiencing these profoundly altered states of consciousness occasioned by psilocybin can develop acute reactions of fear, confusion, panic, or paranoia at some point during the session; however, they also report that with the support of the therapeutic milieu these states are generally transient and have been described by participants and therapists as a necessary and ultimately beneficial process (20).

Alternatively, while the MDMA-AT protocol also includes participants spending some periods in an internal process, more of the session tends to be engaging in a process of embodied self-exploration with the therapists. Some participants described vivid mystical-type visions in their MDMA sessions but largely still felt cohesive in their sense of self during those visionary states. Overall, participants described MEs as playing a less prominent role in their healing process as opposed to a more connected processing of trauma, grief, and exploring their situation with increased self-awareness, compassion, and reduced physiological activation. Rather than providing deep lessons through a profoundly altered state of consciousness, it seems in many cases that MDMA allows participants to engage in a deep process of self-exploration and psychotherapy that they may normally be blocked from. In MDMA-AT qualitative studies, participants have regularly expressed the feeling that it is the process of psychotherapy and self-exploration that leads to change, and the MDMA just allows this to happen in a more successful way (19).

### Limitations

Study limitations included the small sample size and the exploratory nature of the parent study (18). Due to the small sample size and limited diversity in the parent study, the subset of participants in this analysis was largely homogeneous demographically: 100% were female identified, 100% had a cancer diagnosis, and five of six participants identified as White/Caucasian. In order to better assess the effectiveness of this treatment, future studies need to prioritize diversity with regards to race, socioeconomic status, gender, ability, and other biopsychosocial identities. Demographic information such as religious affiliation, spiritual beliefs, or attachment history was also omitted from the data collection due to this analysis taking place with de-identified data. One participant expressed interest in becoming an MDMA-AT therapist, which could have contributed to a positive bias with regards to outcomes and experience of the treatment. Additionally, the experimental nature of this treatment and the increase in popular excitement around it could potentially lead to a positive bias among participants or a desire to minimize negative feelings; steps were taken in the interview structure and questions to minimize these effects. Interviewing 10 of 17 available participants and analyzing only six of the eight available interviews may have contributed to selection bias in the study results; however, measures were taken to reduce this possibility including interviewing the first 10 participants to complete the trial and using randomization to choose those interviews to be used for analysis. The subset of interviews only includes one of the five participants who were in the placebo arm of the trial, and represents only 33% of the complete sample; as such, these results cannot be generalized to portray experiences from the entire sample or be attributed to a wider population. This type of study is not designed to be generalized to a wider population or make predictions about how the treatment will perform in larger studies; rather, this qualitative methodology uses in-depth exploration of specific participant experiences to create a starting point of understanding and a basis for further study.

### Conclusion

This study used qualitative methods to explore the subjective experiences of a subset of participants in the first clinical trial of MDMA-AT for treating anxiety related to LTI. Systematic evaluations of semi-structured qualitative interviews revealed major narrative themes in participants' experiences of their treatment and outcomes. All participants in this study described deeply meaningful experiences and new existential perspectives occasioned by MDMA-AT that they feel have led to considerable improvement in mental health symptoms and quality of life. These interviews also highlight possible psychological mechanisms of action for this treatment, and provide a starting point for exploring the similarities and differences among similar treatments for this indication.

The experiences of these six participants provide evidence that MDMA-AT can be beneficial for people experiencing mental health challenges secondary to LTI, even when they have been resistant to traditional treatments. Larger studies will be necessary to determine the success of MDMA-AT across a wider and more diverse population.

## Data availability statement

The raw data supporting the conclusions of this article will be made available by the authors at request. Some data access requests may require approval of the study sponsor.

## Ethics statement

The studies involving human participants were reviewed and approved by John F. Kennedy University IRB. The patients/participants provided their written informed consent to participate in this study. Written informed consent was obtained from the individual(s) for the publication of any potentially identifiable images or data included in this article.

## Author contributions

WB and MM-W conceived of and wrote the bulk of this report, with input from the other authors. PP and LB created the list of references. TS coordinated with clinical trial and completed the qualitative interviews. All authors contributed to coding, data interpretation, editing, and approved the report before submission.

## Funding

Riverpsykhe, INC provided funding for the researchers to work on this study. Riverpsykhe, INC has no stake, financial or otherwise, in the outcomes of this study, and had no involvement in the study design, collection, analysis, interpretation of data, the writing of this article, or the decision to submit it for publication.

## Conflict of interest

Author WB has worked as a consultant for the study sponsor for different clinical trials, including as an Adherence Rater. Author PP began working with MAPS Public Benefit Corp., on different clinical trials after the core work on this study was completed. Measures were taken to reduce any conflicts these relationships may have presented. Authors do not have any steak financial or otherwise in the outcomes of this study or the parent clinical trial. The remaining authors declare that the research was conducted in the absence of any commercial or financial relationships that could be construed as a potential conflict of interest.

## Publisher's note

All claims expressed in this article are solely those of the authors and do not necessarily represent those of their affiliated organizations, or those of the publisher, the editors and the reviewers. Any product that may be evaluated in this article, or claim that may be made by its manufacturer, is not guaranteed or endorsed by the publisher.
