# Supplementary material for: Facing death, returning to life: A qualitative analysis of MDMA-assisted therapy for anxiety associated with life-threatening illness
Source: Front Psychiatry. 2022 Sep 27;13:944849. doi: 10.3389/fpsyt.2022.944849 (PMC9552520; doi:10.3389/fpsyt.2022.944849)
Supplement: Supplementary file 1 [file Data_Sheet_1.docx]

**Appendix A**

**PROTOCOL MDA-1**

**Inclusion and Exclusion Criteria**

**IND #63,384**

**Protocol Amendment 1: April 29, 2015**

**A Randomized, Double-Blind, Placebo-Controlled Phase 2 Pilot Study of MDMA -Assisted**

**Psychotherapy for Anxiety Associated with a Life-Threatening Illness**

**SPONSOR Multidisciplinary Association for Psychedelic Studies**

 Inclusion Criteria:

1. Diagnosed with life-threatening cancer or non-dementing neurological illness, which can be ongoing or in remission, but with a possibility of recurrence

2. Prognosis of at least nine months life expectancy from the time of screening;

3. Have anxiety due to your illness;

4. Are at least 18 years old;

5. Must be generally healthy;

6. Must sign a medical release for the investigators to communicate directly with their therapist and doctors;

7. Are willing to refrain from taking any psychiatric medications during the study period;

8. Willing to follow restrictions and guidelines concerning consumption of food, beverages, and nicotine the night before and just prior to each experimental session;

9. Willing to remain overnight at the study site;

10. Agree to have transportation other than driving themselves home or to where they are staying after the integrative session on the day after the MDMA session;

11. are willing to be contacted via telephone for all necessary telephone contacts;

12. Must have a negative pregnancy test if able to bear children, and agree to use an effective form of birth control;

13. Must provide a contact in the event of a participant becoming suicidal;

14. Are proficient in speaking and reading English;

15. Agree to have all clinic visit sessions recorded to audio and video

16. Agree not to participate in any other interventional clinical trials during the duration of this study.

Exclusion Criteria:

1. Are pregnant or nursing, or if a woman who can have children, those who are not practicing an effective means of birth control;

2. Weigh less than 48 kg;

3. Are abusing illegal drugs;

4. Are unable to give adequate informed consent;

5. Upon review of past and current drugs/medication must not be on or have taken a medication that is exclusionary.

6. Upon review of medical or psychiatric history must not have any current or past diagnosis that would be considered a risk to participation in the study.

**Appendix B**

**Semi-Structured Interview Guide**

Introduction of interviewer

Hello, my name is _____________________________, and I have been asked to interview you today.

During the interview, I would like to discuss the following topics: the general nature of your experience, as well as subjects that may have arisen during the experience itself, such as autobiographical content, emotional experiences, insight and meaning, spiritual and/or religious experiences, aspects of the experience concerning anxiety related to life-threatening illness, and your relationship with the co-therapist team. The interview should last from and hour and a half to two hours and will be recorded with audio and video equipment. Please feel free to stop at any time for bathroom breaks, and if any questions arise.

| **Interview Guide** | **Clarification questions** |
| --- | --- |
| **Pre-Session** | • Can you expand a little on  this?  • Can you tell me anything  else?  • Can you provide some  examples? |
| Can you tell me your name, age, what you do for work, and what was your diagnosis? |  |
| Can you give me a sense of what life was like prior to the treatment sessions? (probing nature of anxiety and living with their illness, but open ended) |  |
| What led you to participating in the study? What were you hoping for or looking for? |  |
| What were your expectations? |  |
|  |  |
| **The Treatment Session**  Thinking back on your three sessions with MDMA, was there any one session that seems most significant or influential? |  |
| Can you describe in detail your experiences during (this/these) treatment session(s)? |  |
| What emotions arose during your experiences?  What memories arose during your experience? |  |
| What insights or new understandings did you gain? |  |
| What internal changes did you experience? Any perceptual changes, or images? |  |
| How would you describe your experiences after the session ended and in the days following. |  |
| Can you recall any memorable experiences from the other MDMA or non-MDMA sessions? |  |
|  |  |
| **Post-Session Questions** |  |
| In what ways do you feel the study has affected your life since the sessions? |  |
| Thus far, what ways has do you feel your participation in the study has influenced your anxiety?  Are there ways in which you feel the study has not yet felt helpful? |  |
| How has the experience changed your perception of or attitude toward the (illness)? |  |
| In your experience, how do you think MDMA assisted psychotherapy compares to other treatments or therapies you’ve tried? Do you have a sense of how MDMA works to create such changes? |  |

| **Other Questions** | • Can you expand a little on  this?  • Can you tell me anything  else?  • Can you provide  some examples? |
| --- | --- |
| What was your experience of the therapists and how did they affect your sessions? |  |
| Is there anything you wish had been different? |  |
| If given an opportunity, would you repeat the experience? |  |
| Is there anything else that you would like to share or anything you would like to ask me? |  |

**Appendix C**

**Code Book with Descriptions**

MN: Methodological notes (comments for fellow analysts/thoughts about process or methodology)

TN: Theoretical Notes (comments for fellow analysts pertaining to theoretical concepts, including a unit that exemplifies a hypothesis about the treatment)

P?: Puzzlements/questions (Portions of transcript that coders wish to discuss in meeting)

Q: Quotable sentences (a favorite, starred, or important pull quote)

SR: Subject recommendation (example: subject requesting additional MDMA session in treatment)

CS: Censorship by interview subject (limiting what they would say in the interview)

Tx(+/-): References to/Descriptions of MDMA-assisted psychotherapy: treatment as whole

T(+/-): References to/Descriptions of therapists in study

Th(+/-): References to/Descriptions of the therapy portion of the treatment, including therapy techniques, specific aspects of the therapy, or referring specifically to a therapy modality.

M(+/-): References to/Descriptions of the MDMA portion of the treatment

PTx(+/-): References to/Descriptions of psychological treatments other than the study treatment

MedTx(+/-): References to/Descriptions of medical treatments

Rx(+/-): References to/Descriptions of Prescribed Substance use (Any medication prescribed to subject)

NRx(+/-): References to/Descriptions of non-prescribed substance use (any illicit substance use, including non-prescribed rx use)

PSx(+/-): References to/Descriptions of psychological symptoms

MSx(+/-): References to/Descriptions of medical symptoms

NPSx: Narrative of psychological symptoms (Providing more context or consideration than simply referencing or describing the symptom. Example: Making sense of where the symptoms are coming from, or how they impact their life

NMSx: Narrative of medical symptoms (Providing more context or consideration than simply referencing or describing the symptom. Example: Making sense of where the symptoms are coming from…)

MTx(+/-): Motivation (+) or Lack of motivation (-) to participate in any treatment, including any healthy activity that could be a benefit to the person’s overall health, (ex: therapy, Yoga, …)

EL(+/-): Enacting Lessons from the treatment- A description of the participant utilizing a skill or piece of knowledge gained in the study

PEM(+/-) Perceptions/Expectations about MDMA

PET(+/-): Perceptions/Expectations of other treatments

AC(+/-): References to/Descriptions of actions (+/-) a person is taking in their life (ex: attempting suicide (-) or starting a support group (+))

C(+/-): References to/Descriptions of coping skills used- anything the person used in attempt to manage sxs or avoid discomfort (ex: substance abuse (-), mindfulness exercises (+))

S(+/-): Spirituality/religion (An experience involving spiritual or religious artifacts)

PL(+/-): Perception of life (Overarching views on how the world or life works, or how they see the construct of life) Example:  “life is supposed to be happy,” “The world is a cold dark place”

PD(+/-): Perception of death (views around death and dying and if the participant says

“death”) “Death is not something to be afraid of”

Ex(+/-): Existential (relating to a person’s experience of existing/questioning relationship to life or death) Pertains more to the individual’s orientation towards life and death or a direct experience of life and death.   “I am not afraid to die.” “I feel a huge responsibility to live.”

ME(+/-): Mystical experience (non-normal states of being, Noetic and ineffable experiences)

SA(+/-): Self Awareness(+)/judgments about self(-)

IP(+/-):  References to/Descriptions of Interpersonal Relationships, also qualify when possible:

         [SO]: Significant Other

         [FM]: Family

         [FR]: Friends

         [SC]: Society

[PR]: Providers

*For all pertinent codes add these suffixes whenever possible, as appropriate:

- + or - (perceived benefits/concerns or detriments) or (positive/negative references)
- Timeframe notations: add to codes whenever timeframe can be deciphered from statement

(PT): Pre-Treatment (any time prior to study)

(DT): During-Treatment (during the study period)

(ST): Short-Term (Within first month after Final MDMA session)

(LT): Long-Term (More than one month after final MDMA session to the present)
